# Intellectual property protection, legal awareness, and startup growth: evidence from Somalia

**DOI:** 10.3389/fsoc.2026.1900259

**Published:** 2026-10-14

**Authors:** Osman Aded Osman, Adnan Abdukadir Ahmed, Mohamed Liban Isak, Fahad Mohamed Hussein

**Affiliations:** 1 Faculty of Law, SIMAD University, Mogadishu, Somalia; 2 Faculty of Management Sciences, SIMAD University, Mogadishu, Somalia; 3 Faculty of Economics, SIMAD University, Mogadishu, Somalia

**Keywords:** entrepreneurship, intellectual property protection, legal awareness, PLS-SEM, Somalia, startup growth

## Abstract

**Introduction:**

Intellectual property (IP) protection is increasingly recognized as a strategic mechanism through which startups can safeguard innovation, strengthen competitive advantage, and support business development. However, limited empirical evidence exists regarding the effectiveness of IP protection in fragile institutional environments such as Somalia. This study examines the effects of intellectual property protection and legal awareness on startup growth and investigates whether legal awareness strengthens the relationship between IP protection and growth.

**Methods:**

This study employed quantitative cross-sectional survey design. Data were collected through a structured questionnaire from 220 startup founders, owners, managers, and senior employees in Somalia. Partial Least Squares Structural Equation Modelling (PLS-SEM) was used to assess the measurement model and test the direct and moderating relationships among intellectual property protection, legal awareness, and startup growth.

**Results:**

The findings demonstrate that intellectual property protection has a positive and significant relationship with startup growth. Legal awareness also positively influences startup growth and emerges as the stronger of the two direct predictors. However, legal awareness does not significantly moderate the relationship between intellectual property protection and startup growth. These findings indicate that legal awareness operates primarily as an independent growth-enabling capability rather than as a condition that strengthens the growth effect of intellectual property protection.

**Discussion:**

The study extends entrepreneurship and intellectual property research by providing empirical evidence from a fragile and under-researched institutional context. The findings suggest that startup development depends not only on protecting intangible assets but also on strengthening entrepreneurs’ legal capabilities. The study therefore highlights the importance of integrating practical legal awareness, IP management, and accessible legal support into entrepreneurship education, incubation programs, and startup policy in developing economies.

## 1 Introduction

The role of startups as key players in innovation-driven economic growth has become more prominent than ever before, as they bring new products, services, technologies and business models to markets that are typically dominated by large, established companies. During this process, intangible assets like trademarks, copyrights, patents, industrial designs, trade secrets, software, brand identity and business know-how are becoming vital competitive assets. IP protection allows startups to capture the rewards of innovation, mitigate the threat of imitation, build market identity, and convey legitimacy to customers, investors, and partners. Recent research also indicates that IP management is not just a legal matter, but a strategic business capability that can enhance innovation performance, competitiveness, financing, and long-term growth (Amankwah-Amoah and Medase, 2023).

The relationship between intellectual property protection and startup growth is important but not always direct. IP protection can help startups grow by safeguarding unique assets, enhancing market credibility, enabling licensing opportunities, and boosting investor confidence. But it can have different impacts on IP types, sectors, institutional contexts, and firm capabilities. Power and Reid (2021) found that trademarks and outward licensing can enhance startup performance, and patents cannot always directly benefit young firms. Likewise, Rieger et al. (2025) demonstrated that trademark applications can serve as early legitimacy signals for VC seed funding, and (Taglialatela and Barontini, 2024) reported that patent applications can be linked to sales improvement but not profitability. The results suggest that IP protection is not a self-evident accelerator of startups’ growth but rather a tool that can be used strategically, depending on the knowledge, management and exploitation of the legal rights of the entrepreneurs.

In emerging and developing economies, the protection of intellectual property is particularly important as startups are typically in an environment of low institutional support, limited access to finance, high informality, and low awareness of legal protection mechanisms. Research in sub-Saharan Africa and other emerging markets reveals that IP can contribute to innovation intensity, sales growth, technological innovation and sustainable innovation performance, provided that the institutions are of high quality, the management is capable, innovation collaboration is present, and complementary resources are available (Odei, 2024). The findings from Ghana and other sub-Saharan countries also suggest that trademarks, copyrights, industrial designs, and patents can have an impact on innovation and firm performance, but the impact varies by firm and type of protection (Amankwah-Amoah and Medase, 2023).

Somalia is a unique context for this study as the startup ecosystem is developing in a fragile institutional and legal landscape. Somalia has recently demonstrated entrepreneurial momentum, with signs that youth entrepreneurship is a key driver of income generation, service provision and local economic resilience. Limited formal employment, weak institutional capacity, limited access to finance, and a narrow industrial base are among the factors that influence youth entrepreneurship in Somalia, according to the [Somalia National Bureau of Statistics (SNBS), 2024] from the 2024 Business Establishment Census.

According to the *Global Startup Ecosystem Index 2025* produced by Startup Blink, Somalia re-entered the global top 100 startup ecosystems, ranking 100th globally and 4th in Eastern Africa. Response Innovation Lab, Startup Blink’s ecosystem partner in Somalia, further reported that Somalia ranked 13th among African startup ecosystems in the 2025 index (StartupBlink, 2025).

Startup development may generate benefits beyond firm-level performance, including employment creation, greater access to goods and services, technological innovation, and solutions to social and developmental challenges. However, commercial growth should not automatically be equated with broader public value. Research conducted within the Sustainable Development Goals framework shows that entrepreneurial organizations differ considerably in the extent to which their commercial activities contribute to social objectives, inclusion, sustainability, and public problem-solving (Li et al., 2024). This distinction is particularly relevant in developing economies, where the contribution of startups should ultimately be considered not only in terms of firm growth but also in relation to employment quality, accessibility and affordability of products and services, and wider societal benefits.

Despite this entrepreneurial development, however, Somalia’s IP environment is still institutionally developing, and enforcement capacity is still limited. According to official information, the Ministry of Commerce and Industry is responsible for the registration of trademarks in Somalia, and the purpose of registration is to safeguard the identity of the brand, to avoid consumer confusion and to provide the trademark owner with exclusive rights over the trademark (Federal Republic of Somalia, 2026). Professional legal analysis reveals, however, that Somalia does not have a common-law IP system, that trademark and copyright rights are primarily statutory, that copyright protection is often contractual and based on proof of authorship, and that Somalia does not currently have specialized intellectual property courts (Chambers and Partners, 2026). Furthermore, the U.S. International Trade Administration reports that Somali courts are not well equipped to resolve IP claims, due to the weak judicial environment. These conditions suggest that Somali startups face both formal legal limitations and practical enforcement challenges (International Trade Administration, 2024).

Legal awareness is thus a key concern in the Somali startup environment. While many startups are increasingly relying on intangible assets, entrepreneurs may not have the legal knowledge needed to effectively identify, protect, manage and commercialize these assets. Formal IP protection alone can be a driver of growth only in fragile institutional environments where entrepreneurs are aware of the relationship between IP rights and branding, licensing, contracting, market entry, investor negotiation, digital platforms, and dispute prevention. Despite the importance of IP for competitiveness and successful firm development, recent studies indicate that IP awareness and IP strategy are not well integrated into SME business strategy (Khawand et al., 2024). The study on legal awareness and MSME compliance also indicates that legal knowledge can have an impact on the behavior of businesses, compliance with regulations, and the capacity of small businesses to function within formal institutional systems (Sukania et al., 2025).

While the literature has explored intellectual property protection, innovation, SME performance and startup financing, there are some important research gaps. First, existing IP–startup studies focus on developed economies or more robust institutional settings, with fragile and post-conflict economies being underrepresented (Lohrey and Willoughby, 2025). Second, although IP and innovation performance have been studied in the context of African studies, little empirical research has been conducted on the growth of startups in fragile economies like Somalia (Adomako and Tran, 2025). Third, an important unresolved issue concerns the role of legal awareness in determining whether formal intellectual property protection can be translated into meaningful business outcomes. Existing studies generally examine IP awareness, utilization, or legal knowledge as direct predictors of innovation, compliance, or firm performance, while comparatively little attention has been given to whether legal awareness changes the strength of the relationship between intellectual property protection and startup growth (Khawand et al., 2024; Sukania et al., 2025). Examining this conditional relationship is important because possessing an IP right does not necessarily imply that an entrepreneur can effectively identify ownership risks, negotiate licensing or contractual arrangements, prevent infringement, or enforce legal rights. This issue is particularly urgent in institutionally fragile environments, where formal legal mechanisms may exist, but their accessibility, enforceability, and practical utility remain constrained. Somalia therefore provides a theoretically important setting in which to examine whether entrepreneurs’ legal awareness enables them to derive greater growth value from intellectual property protection.

Against this background, this study examines the direct effects of intellectual property protection and legal awareness on startup growth and tests whether legal awareness conditions the relationship between intellectual property protection and growth among startups in Somalia. The study makes three principal contributions. First, it extends the intellectual property and entrepreneurship literature by providing firm-level empirical evidence from Somalia, a fragile and under-researched institutional context that remains largely absent from existing IP–startup research. Second, it advances the conceptual treatment of legal awareness by examining it not only as a direct entrepreneurial capability but also as a potential boundary condition that may influence the extent to which startups convert protected intangible assets into growth outcomes. This enables a more nuanced test of Resource-Based View, Resource Orchestration Theory, and Institutional Theory in an environment where legal protection and institutional enforcement are uneven. Third, the study provides practical value for startup founders, managers, incubators, legal professionals, and policymakers by identifying whether startup development depends primarily on formal IP protection, entrepreneurs’ legal capabilities, or the interaction between the two. Such evidence can inform entrepreneurship education, legal-support services, IP registration initiatives, and institutional reforms designed to strengthen startup ecosystems in developing and fragile economies.

The research investigates three main hypotheses. First, it explores the positive impact of IP protection on startup growth. Second, it investigates the positive impact of legal awareness on the growth of startups. Third, it examines whether the relationship between IP protection and startup growth is moderated by legal awareness. The proposed conceptual framework thus places legal awareness as a boundary condition, which either enhances or diminishes the value of IP protection for startup growth. This model is methodologically suitable because the relationships proposed in this model include latent constructs and a conditional effect that can be studied by Structural Equation Modelling. In the recent literature of PLS-SEM, the importance of testing complex models, moderation effects, predictive relationships, and theoretically justified boundary conditions in business and management research has been highlighted (Hair et al., 2024).

The remainder of the paper is organized as follows. Section 2 presents the Literature Review and Contingent Hypotheses, including theoretical foundations, relevant empirical evidence, and development of the study hypotheses. Section 3 describes the Data and Method, including the research design, sampling strategy, data collection procedures, measurement instruments, and analytical strategy. Section 4 presents the Results, including the respondent profile, descriptive statistics, measurement-model assessment, discriminant validity, and structural-model findings. Section 5 discusses the findings in relation to the theoretical framework and existing literature and considers their theoretical and practical implications. Finally, Section 6 presents the Conclusion, summarizes the main findings, acknowledges the study limitations, and outlines directions for future research.

## 2 Literature review and contingent hypotheses

### 2.1 Theoretical foundations

This study integrates the Resource-Based View (RBV), Resource Orchestration Theory, and Institutional Theory to explain how intellectual property protection and legal awareness may influence startup growth in Somalia. These perspectives are complementary rather than competing. RBV explains why protected intangible assets may constitute strategically valuable firm resources; Resource Orchestration Theory explains why managerial capabilities are necessary to deploy and derive value from those resources; and Institutional Theory explains how the effectiveness of resources and capabilities is shaped by the wider legal, regulatory, and institutional environment. Integrating these perspectives provides a multilevel explanation linking firm resources, managerial capabilities, and institutional conditions to startup growth.

The Resource-Based View provides the first theoretical foundation of the study. RBV argues that differences in firm performance can arise from heterogeneity in strategically important resources and that resources capable of generating sustained competitive advantage are generally valuable, rare, difficult to imitate, and difficult to substitute (Barney, 1991). In the startup context, intangible assets such as trademarks, copyrights, software, trade secrets, designs, brand identity, business know-how, and proprietary knowledge may constitute strategically valuable resources because they can support differentiation, reduce imitation, strengthen legitimacy, and enable firms to appropriate returns from innovation. Intellectual property protection can enhance the strategic value of these assets by strengthening a startup’s ability to establish ownership, control their use, commercialize them, and capture economic value. RBV therefore provides the principal theoretical explanation for the proposed direct relationship between intellectual property protection and startup growth.

However, possessing valuable resources does not by itself explain how firms convert those resources into superior performance. Resource Orchestration Theory extends resource-based reasoning by explicitly emphasizing managerial actions through which resources are structured, bundled, and leveraged to create value (Sirmon et al., 2011; D’Oria et al., 2021). Sirmon et al. specifically identify structuring, bundling, and leveraging as central managerial processes through which resources are converted into competitive advantage. From this perspective, legal awareness can be conceptualized as an entrepreneurial capability that enables founders and managers to recognize protectable assets, understand ownership rights, use contractual and confidentiality safeguards, negotiate licensing arrangements, identify infringement risks, and seek appropriate legal assistance. A startup may therefore possess valuable IP-related resources without fully realizing their strategic value when its decision-makers lack the knowledge required to organize and deploy them effectively. Resource Orchestration Theory consequently provides the theoretical basis for treating legal awareness both as a direct growth-related capability and as a potential complementary capability that could strengthen the value derived from intellectual property protection.

Institutional Theory provides the third theoretical foundation by recognizing that organizational behavior and economic outcomes are embedded within institutional environments. Institutions establish formal and informal constraints that structure economic interaction and shape the opportunities available to firms (North, 1990), while organizational institutional theory emphasizes that regulative, normative, and cultural-cognitive arrangements influence organizational behavior, legitimacy, and access to resources (Scott, 2014). The regulative dimension is particularly relevant to this study because intellectual property rights depend on laws, registration procedures, enforcement mechanisms, courts, regulatory agencies, and other formal institutions. In institutionally stronger environments, firms may be able to register, commercialize, license, and enforce intellectual property rights relatively predictably. In institutionally weaker environments, formal rights may exist while their practical accessibility, enforcement, and commercial utilization remain constrained. This is particularly relevant in Somalia, where trademark registration mechanisms are available, but specialized IP adjudication and broader enforcement capacity remain limited (International Trade Administration, 2024; Chambers and Partners, 2026). Institutional Theory therefore explains why the growth value of intellectual property protection and legal awareness may differ from that observed in more developed institutional environments.

Taken together, the three theoretical perspectives provide a more complete explanation of the proposed model than any single theory could offer. RBV identifies protected intangible assets as potentially valuable strategic resources; Resource Orchestration Theory explains how entrepreneurs’ legal awareness may enable them to organize, deploy, and exploit those resources; and Institutional Theory explains how the usefulness of both IP protection and legal capabilities is conditioned by the surrounding institutional environment. The integration is particularly important in Somalia because startups may possess valuable intangible assets and legal knowledge while operating in an environment where the enforceability and commercial usability of formal rights remain uneven. The framework therefore links resources (IP protection), managerial capability (legal awareness), institutional conditions, and performance (startup growth) and provides the theoretical basis for the direct and contingent hypotheses developed below.

### 2.2 Intellectual property protection and sustainable development: benefits and trade-offs

The relationship between intellectual property protection and sustainable development is inherently dual-sided. On the one hand, intellectual property rights can provide economic incentives for firms, researchers, and entrepreneurs to invest in research and development by enabling innovators to appropriate part of the economic returns generated by their inventions and creative outputs. Such incentives can be particularly important in technology-intensive sectors where innovation requires substantial upfront investment and where imitation may otherwise reduce the expected returns from innovative activity. From a Sustainable Development Goals perspective, effective intellectual property systems may therefore contribute to technological innovation, entrepreneurship, and industrial development associated with SDG 8 on decent work and economic growth and SDG 9 on industry, innovation, and infrastructure. They may also encourage investment in technologies relevant to public health, clean energy, climate mitigation, agriculture, and digital transformation. World Intellectual Property Report 2024 recognizes intellectual property as an important mechanism for encouraging innovation and creativity relevant to achieving the SDGs (Oriakhogba et al., 2025).

However, stronger intellectual property protection does not necessarily generate uniformly positive developmental outcomes, particularly in developing and least-developed economies. Intellectual property rights confer temporary exclusivity, and this exclusivity can increase the costs of obtaining, reproducing, adapting, or disseminating protected technologies. The resulting trade-off is particularly visible in areas where innovations have substantial public-good characteristics. In public health, for example, patents may support investment in pharmaceutical research while simultaneously raising concerns about the affordability and accessibility of medicines. The World Trade Organization’s Doha framework therefore recognizes both the role of intellectual property in encouraging pharmaceutical innovation and the need for policy flexibilities, including compulsory licensing, where necessary to protect public health and facilitate access to medicines. Similar tensions arise in relation to climate technologies, where strong protection may encourage private investment while licensing costs or restrictions on follow-on innovation may constrain the diffusion and adaptation of technologies required for climate mitigation and sustainable development in lower-income economies (World Trade Organization, 2001).

Recent research consequently challenges the assumption that progressively stronger intellectual property protection automatically produces greater innovation in developing economies. Khouilla and Bastidon (2024), in a review of the literature on intellectual property protection and technological catch-up, find that the relationship between intellectual property protection and innovation is nonlinear and highly dependent on a country’s level of technological and institutional development. Their review indicates that the benefits of stronger protection tend to be more evident in advanced economies, whereas countries at lower levels of technological development may depend more heavily on knowledge diffusion, imitation, learning, and adaptation. In such environments, excessively stringent protection may restrict opportunities for technological catch-up rather than accelerate them.

The same tension is evident in climate-related innovation. Jee et al. (2026) show that intellectual property rights can encourage investment and facilitate certain forms of international technology transfer, but they can also raise the costs of accessing foreign technologies and restrict adaptive innovation in developing countries. Their findings emphasize that the developmental effects of intellectual property depend not simply on the strength of legal protection but also on domestic technological capabilities, access to finance, market conditions, institutional capacity, and the ability of local firms to absorb and adapt technology. Recent World Intellectual Property Report 2024 analysis similarly stresses that intellectual property rights can incentivize innovation while also influencing the speed and distribution of technology diffusion, thereby requiring policymakers to balance incentives for creation with broader access and dissemination objectives (World Intellectual Property Organization, 2024).

These competing perspectives are particularly relevant to Somalia. A stronger intellectual property framework could help Somali startups protect brands, software, creative products, technological solutions, and other intangible assets, thereby encouraging investment and entrepreneurial experimentation. At the same time, Somalia remains a developing and institutionally fragile economy in which firms often depend on imported technologies, knowledge transfer, adaptation, and relatively affordable access to external innovations. Consequently, strengthening IP protection should not be understood as an objective. Rather, the appropriate policy challenge is to develop an intellectual property system that protects legitimate entrepreneurial innovation while preserving opportunities for knowledge diffusion, technology adaptation, competition, public access, and socially beneficial innovation. From an SDG perspective, the developmental value of IP protection therefore depends on achieving an appropriate balance between private incentives and wider societal interests.

In this regard, this study considers intellectual property protection as a strategic resource, legal awareness as a managerial and legal capability, and startup growth as the performance outcome. This integrated theoretical approach is suitable for SEM because the proposed model includes latent constructs, direct effects, and a moderation effect. It also addresses recent calls for IP research that is more focused on SMEs, models of entrepreneurship that are more context-sensitive, and more empirical testing of the impact of intangible resources on performance in developing and institutionally fragile contexts (Amankwah-Amoah and Medase, 2023).

While most of the recent SEM studies have been carried out outside Somalia, they offer valuable evidence for the study of intellectual property protection, innovation and business outcomes. Chen et al. (2024) used PLS-SEM and artificial neural network modelling to examine the role of intellectual property protection in radical technological innovation among 336 members of national research project teams in China. Similarly, Yin et al. (2024) examined IP protection as a driver of radical technological innovation through innovation milieu and group potential. These studies demonstrate the usefulness of SEM for modelling IP protection and innovation mechanisms, but they do not examine startup growth, legal awareness, or fragile institutional contexts in Africa.

Other SEM-PLS evidence shows that IP awareness, IP utilization and IP protection can impact innovation, competitiveness and MSME growth. The findings of the SEM-PLS study on the MSMEs in Indonesia indicated that there was a positive relationship between IP awareness and IP utilization, a strong relationship between IP protection and innovation, and a significant effect on competitiveness. This is important because it connects IP-related knowledge to business outcomes, but it is not the same as the current study as it is conducted with MSMEs in Indonesia instead of startups in Somalia, and it does not include legal awareness as a moderator. Based on the results of this study, it is appropriate to examine IP protection, legal awareness, and startup growth within one SEM framework (Chen et al., 2024).

The quantitative literature as a whole suggests that the IP–performance relationship is not necessarily linear. Power and Reid (2021) found that various types of IP have varying impacts on startup performance in a longitudinal panel of U.S. startups. Amankwah-Amoah and Medase (2023) reported that the impact of IP on performance is different in sub-Saharan Africa, depending on the type of IP (patents, copyrights, industrial designs, and trademarks). Grimaldi et al. (2021) also demonstrates that companies have varying IP strategies, such as defensive, collaborative, and impromptu approaches, suggesting that IP protection can have a greater impact when it is managed strategically and not just as a formal legal activity. Likewise Yin et al. (2024) demonstrate how IP protection can facilitate radical technological innovation via intermediate mechanisms.

Somalia’s startup ecosystem has gained greater regional visibility. In the *Global Startup Ecosystem Index 2025*, Somalia re-entered the world’s top 100 startup ecosystems, ranking 100th globally and 4th in Eastern Africa; Response Innovation Lab additionally reported that Somalia occupied 13th position in Africa in the same 2025 ranking (Response Innovation Lab, 2025). Concurrently, the institutional framework of Somalia’s IP system is weak, as trademark protection is based on registration, copyright registration is limited, and IP litigation is conducted in ordinary civil courts instead of specialized IP courts. Somalia is a theoretically relevant context to test the relationship between IP awareness and entrepreneurial growth, due to the entrepreneurial growth potential and institutional fragility (Chambers and Partners, 2026).

### 2.3 Intellectual property protection and startup growth

Intellectual property protection can influence startup growth by enabling firms to appropriate returns from innovation, differentiate their offerings, protect brand identity, reduce imitation, and strengthen their legitimacy in the eyes of customers, investors, and commercial partners. From the Resource-Based View, protected intangible assets may constitute strategically valuable resources that enhance competitive advantage when they are difficult to imitate or substitute. Empirical evidence supports this argument, although the strength of the relationship varies across different forms of intellectual property and institutional environments. Power and Reid (2021), for example, show that intellectual property instruments can influence startup performance, while Grimaldi et al. (2021) emphasize that the benefits of intellectual property depend substantially on how firms strategically manage and exploit protected knowledge. Evidence from sub-Saharan Africa similarly indicates that intellectual property can support innovation and firm-level performance, although its effects vary according to the form of protection and the institutional context (Amankwah-Amoah and Medase, 2023).

The Somali context provides additional justification for examining this relationship. Official and grey literature indicates that Somalia has a developing intellectual property regime in which trademark registration is formally available, while broader enforcement capacity remains constrained by institutional and judicial limitations (International Trade Administration, 2024). At the same time, Somalia’s startup ecosystem has expanded across digital services, e-commerce, fintech, education, media, and other sectors in which intangible assets such as brands, software, creative content, designs, and business know-how are economically important. In such an environment, the ability of startups to protect these assets may reduce imitation risks, reinforce market identity, and enhance their capacity to expand. Accordingly, the study proposes:

H1: Intellectual property protection has a positive effect on startup growth in Somalia.

### 2.4 Legal awareness and startup growth

Legal awareness may also represent an important entrepreneurial capability because startups operate within contractual, regulatory, ownership, licensing, confidentiality, and dispute-resolution frameworks. Resource Orchestration Theory suggests that resources alone do not automatically produce performance outcomes; managers must possess the knowledge and capabilities required to structure, combine, and leverage those resources effectively (D’Oria et al., 2021). Applied to the present study, entrepreneurs who understand intellectual property registration, ownership rights, contracts, licensing arrangements, infringement risks, confidentiality obligations, and available legal remedies may be better positioned to protect business relationships, reduce uncertainty, and make informed strategic decisions.

Existing research supports the importance of legal and intellectual property knowledge for SMEs. Khawand et al. (2024) show that intellectual property knowledge and strategy are important components of SME decision-making, while Sukania et al. (2025) link legal protection and awareness to SME performance and compliance. These issues are particularly relevant in Somalia because entrepreneurs operate in a legal environment where formal intellectual property mechanisms coexist with significant enforcement and institutional constraints. The International Trade Administration (2024) reports limitations in the capacity of Somali courts to address intellectual property disputes, while Chambers and Partners (2026) notes the absence of specialized intellectual property courts. Under such conditions, legal awareness may be especially valuable because entrepreneurs may need to rely more heavily on preventive measures, documentation, contractual safeguards, and informed decision-making. Therefore:

H2: Legal awareness has a positive effect on startup growth in Somalia.

### 2.5 The moderating role of legal awareness

Although intellectual property protection may provide startups with valuable legal and strategic resources, the benefits of those resources may depend on entrepreneurs’ ability to understand and use them effectively. Resource Orchestration Theory provides a basis for this conditional argument because firm resources generate greater value when managers possess complementary capabilities that enable their effective deployment. Legal awareness may therefore strengthen the growth benefits of intellectual property protection by enabling startup founders and managers to recognize protectable assets, understand registration and ownership requirements, negotiate licensing and contractual arrangements, manage infringement risks, and identify appropriate enforcement mechanisms.

This expectation is also consistent with Institutional Theory. The effectiveness of intellectual property rights depends partly on the broader legal and regulatory environment in which firms operate.

In institutionally fragile settings, formal protection may exist without being easily enforceable or commercially exploitable. Somalia represents such a context: official and professional sources indicate the availability of certain formal intellectual property mechanisms alongside continuing limitations in enforcement, specialized adjudication, and legal infrastructure (International Trade Administration, 2024; Chambers and Partners, 2026). Under these circumstances, entrepreneurs with stronger legal awareness may be better able to navigate institutional constraints and derive greater strategic value from the intellectual property protections available to them.

Previous research has generally examined intellectual property awareness, protection, and utilization as direct determinants of innovation, competitiveness, or firm performance, while comparatively little research has tested whether broader legal awareness conditions the relationship between intellectual property protection and startup growth. This creates an important empirical gap, particularly in fragile and under-researched entrepreneurial environments. Accordingly, the study proposes:

H3: Legal awareness positively moderates the relationship between intellectual property protection and startup growth, such that the positive effect of intellectual property protection on startup growth is stronger when legal awareness is high.

### 2.6 Inconsistencies and knowledge gaps

The first gap is the mixed impact of IP protection on firms’ outcomes. The impact of IP protection on innovation, competitiveness and performance has been demonstrated in some studies, but not in others, and is dependent on the nature of the IP, the sector and the way in which the IP rights are used. For instance, Power and Reid (2021) demonstrate that the impact of various IP instruments varies; Amankwah-Amoah and Medase (2023) find that there is variation in the forms of IP; and Grimaldi et al. (2021) discover how IP strategy varies depending on the defensive, collaborative or unclear stance that companies adopt toward IP protection. The inconsistencies indicate that IP protection should not be assumed to be a direct driver of growth.

The second gap is contextual. Most of the SEM and quantitative studies on IP protection and innovation and business performance have been conducted in China, Indonesia, the United States or in larger sub-Saharan African samples. Somalia’s fledgling start-up scene and its precarious legal-institutional landscape are largely absent from empirical IP–start-up research. This is significant as theories that are effective in stronger institutional settings may not be as effective in weaker institutional settings, where IP courts are not in place, and entrepreneurs may not be aware of formal IP mechanisms (Chen et al., 2024; Chambers and Partners, 2026). The third gap is related to the role of legal awareness. Past studies tend to look at IP awareness or IP utilization as direct drivers of innovation, competitiveness or compliance. However, there are less studies that consider legal awareness as a moderator that influences the usefulness of IP protection for startups’ growth. This is important in theory, as a startup could have IP rights but not be able to use them if the founder is unaware of the risks of registration, ownership, licensing, enforcement or infringement. Recent evidence suggests that IP awareness affects IP utilization and MSME competitiveness, while Somali legal guidance shows that IP protection is based on registration, documentation, contractual agreements and ordinary court enforcement (Chambers and Partners, 2026). Based on these gaps, this study develops a moderation-based SEM model. The model proposes that IP protection has a direct effect on startup growth and tests whether legal awareness strengthens this relationship. The model extends RBV by emphasizing the importance of intangible legal resources for growth, resource orchestration theory by emphasizing the importance of legal awareness in enabling entrepreneurs to leverage these resources, and institutional theory by testing the model in the fragile legal and entrepreneurial environment of Somalia (Aba Sey et al., 2010; Hair et al., 2024; D’Oria et al., 2021).

## 3 Data and method

### 3.1 Research design

This study employed a quantitative cross-sectional survey design to examine the relationships among intellectual property protection, legal awareness, and startup growth in Somalia. A cross-sectional design was appropriate because data were collected from startup respondents at one point in time to test the proposed hypotheses. The use of SEM was also suitable because the main constructs intellectual property protection, legal awareness, and startup growth are latent variables measured through multiple indicators rather than single observed items (Creswell and Creswell, 2018).

The study was explanatory because it tested theory-based hypotheses on the direct effects of intellectual property protection and legal awareness on startup growth. It was also predictive because PLS-SEM was used to assess the extent to which the model explained variance in startup growth. This design was appropriate because the model included latent constructs, direct relationships, and a moderation effect (Hair et al., 2019).

### 3.2 Target population

The target population of this study consisted of startup founders, co-founders, owners, managers, and senior employees working in Somalia. These respondents were selected because they are directly involved in business decision-making and are more likely to understand their firms’ IP practices, legal awareness, market position, and growth experience. All respondents were adults and had sufficient knowledge of their startups’ legal, operational, and growth-related activities (Grimaldi et al., 2021; Lohrey and Willoughby, 2025; Power and Reid, 2021).

At the national level, an exact population figure specifically for startups is not currently available because Somalia does not yet maintain a comprehensive startup-specific national registry. The Somalia Integrated Business Establishment Census (SIBEC-2024), conducted by the Somali National Bureau of Statistics, identified 174,149 active business establishments across the country, comprising formal, informal, and micro-establishments (Somali National Bureau of Statistics [SNBS], 2024). However, the census does not separately identify firms according to a standardized startup classification. Consequently, the 174,149 establishments should not be interpreted as the population of startups. For the present study, the target population was therefore defined operationally as founders, co-founders, owners, managers, and senior employees of young entrepreneurial firms operating in Somalia who possessed sufficient knowledge of their firms’ intellectual property practices, legal issues, and growth activities. The absence of a complete startup-specific sampling frame also informed the use of purposive non-probability sampling.

The context of Somalia is crucial and interesting for research, as the startup ecosystem is growing in a weak institutional and legal framework. Somali startups are now operating in a wide range of sectors that are increasingly relying on intangible assets such as digital services, edtech, fintech, telecom, consultancy, retail, media and creative industries. But previous literature shows that the quality of the institutions, managerial skills, innovation cooperation and availability of complementary resources have proven to affect the effectiveness of IP protection, particularly in developing and emerging economies (Adomako and Tran, 2025). This is why Somalia is an appropriate ground to explore the impact of legal awareness on the utility of IP protection for startups’ development.

The study concentrated on the people who had enough information about the working process, legal aspects, and development of their startups. This was needed because the constructs in the model, namely the intellectual property protection, legal awareness and startup growth, call for informed organizational level responses. Knowledgeable respondents will help the data be more relevant and limit the risk of being unable to capture the responses of respondents who are not familiar with the firm’s legal or strategic decisions (Hair et al., 2024).

### 3.3 Sampling strategy and sample size

A purposive non-probabilistic sampling technique was employed to identify respondents of the start-ups who have directly experienced business operations and legal protection practices and have decided about the growth of their businesses. Purposive sampling is appropriate when the researcher is concerned that a particular type of knowledge or experience is required on the phenomenon being studied. The Somali context presents a methodological challenge to obtain a complete and reliable national sampling frame of startups which necessities purposive sampling to reach relevant startup founders, owners, managers, and senior employees (Etikan, 2016; Creswell and Creswell, 2018).

A total of 250 questionnaires were distributed to eligible respondents. Of these, 220 completed questionnaires were returned and retained for analysis, yielding a response rate of 88.0%. The final sample of 220 respondents was used for the measurement and structural model analyses. The sample size was determined based on the requirements of PLS-SEM. The 10-times rule proposes that the minimum number of subjects should be 10 times the maximum number of structural paths connected to one endogenous construct. In this model, three variables, namely, IP protection, legal awareness and interaction between IP protection and legal awareness are predicted to explain the growth of startups. Thus, if the 10-times rule is to be followed, it would suggest that the minimum number of responses required would be 30, and the actual sample of 220 is significantly higher than this.

Additionally, a statistical power consideration was given to the adequacy of the sample. Power analysis is recommended because the 10-times rule is a rough minimum guideline, and does not completely consider effect size, significance level or statistical power. There are several previous studies suggesting a power-based sample size determination for SEM and behavioral research (Faul et al., 2007). A total of 220 respondents were used, which offers sufficient statistical power for testing direct effects, as well as the moderation effect, in the model of the three predictors of start-up growth.

Because purposive non-probability sampling was used, the findings should not be treated as statistically representative of all Somali startups. However, they provide useful analytical insights into startups whose founders, managers, and senior employees have direct knowledge of IP protection, legal awareness, and business growth practices. This restriction is prevalent in applied research in entrepreneurship and management where the exact number of elements in the target population is hard to determine (Etikan, 2016). The use of knowledgeable and appropriate respondents, however, enhances the analytical quality of the data for analysis of the proposed relationship.

### 3.4 Data collection procedure

A pre-structured questionnaire was used to obtain data from startup founders, co-founders, owners, managers and senior employees in Somalia. The three constructs in the study, namely, intellectual property protection, legal awareness and startup growth were measured from the questionnaire. The study necessitated the use of a structured survey due to the need for comparable quantitative responses over a relatively large number of respondents and the requirement of the SEM analysis to have standardized measurement indicators (Sarstedt et al., 2022).

Potential respondents were identified through entrepreneurship networks, business contacts, business communities, and professional networks based on the eligibility criteria established for the study. A total of 250 eligible respondents were approached and invited to participate in the survey. Of these, 220 complete questionnaires were returned and retained for analysis, yielding an overall response rate of 88.0%.

The respondents were briefed on the objectives of the study, the voluntary nature of the respondents to participate in the study, and the confidentiality of their responses before completing the questionnaire. The questionnaire was sent via entrepreneurship networks, business contacts, business communities, and professional networks. The respondents were asked to answer according to their startup’s legal protection practices, their legal awareness and the growth experience of their startup. A total of 250 questionnaires were distributed to eligible startup founders, co-founders, owners, managers, and senior employees through entrepreneurship networks, business contacts, business communities, and professional networks. Of these, 220 completed questionnaires were returned and retained for analysis, representing a response rate of 88.0%. The remaining 30 questionnaires were not returned. The final sample therefore consisted of 220 respondents whose responses were used in the subsequent PLS-SEM analysis.

The questionnaires were screened prior to analysis, to ensure suitable data for multivariate analysis. Completed responses, straight-line answering, inconsistent patterns of responding and missing values were checked for. It is recommended to use data screening prior to SEM because poor quality responses can cause errors in the reliability, validity and path estimates in the structural model. Written informed consent was obtained from all participants before they completed the questionnaire.

### 3.5 Measurement instruments

The survey instrument measured three latent constructs: intellectual property protection (IPP), legal awareness (LA), and startup growth (SG). The questionnaire was developed through a literature-informed adaptation process rather than by reproducing a single existing instrument. The conceptual domains of the three constructs were informed by prior studies on intellectual property protection and strategic IP management, legal and intellectual property awareness, and startup and SME performance. The wording of the items was subsequently contextualized to reflect the legal, institutional, and entrepreneurial characteristics of startups operating in Somalia.

Intellectual property protection was conceptualized as the extent to which startups identify, protect, manage, and strategically use their intangible assets. The measurement domains were informed by previous research examining the role of intellectual property rights and IP strategies in startup and SME performance (Power and Reid, 2021; Grimaldi et al., 2021; Amankwah-Amoah and Medase, 2023). The items addressed the protection and management of relevant intangible assets, including business names, trademarks, logos, software, creative works, trade secrets, and business ideas, as well as formal registration where applicable, contractual and confidentiality safeguards, and the strategic use of IP protection in business development.

Legal awareness was conceptualized as the extent to which startup founders, owners, managers, and senior employees understand the legal mechanisms, rights, procedures, and risks relevant to business protection and development. The measurement domains were informed by literature addressing intellectual property knowledge, legal awareness, and the strategic use of legal protection among SMEs and entrepreneurs (Khawand et al., 2024; Sukania et al., 2025). The indicators covered awareness of IP registration, ownership rights, contractual and licensing arrangements, confidentiality, infringement risks, and access to appropriate legal assistance.

Startup growth was measured using perceptual indicators of business development. The measurement domains were informed by entrepreneurship and intellectual property research examining startup and SME performance (Power and Reid, 2021; Taglialatela and Barontini, 2024). The five indicators captured perceived developments in sales, customer base, market reach, business expansion, and competitive position. Business expansion reflected developments in products or services and operational capacity. Perceptual measures were considered appropriate because many young and privately held startups may not maintain standardized audited financial statements or may be reluctant to disclose commercially sensitive financial information.

IPP, LA, and SG were operationalized as multi-item latent constructs rather than single observed variables. Each construct was measured using five questionnaire indicators, resulting in a total of 15 measurement items. The indicators were coded IPP1–IPP5 for intellectual property protection, LA1–LA5 for legal awareness, and SG1–SG5 for startup growth. The individual items served as observed indicators of their respective latent constructs in the PLS-SEM measurement model. All items were assessed using a five-point Likert scale ranging from 1 = strongly disagree to 5 = strongly agree.

The wording of the indicators was adjusted to ensure contextual relevance to Somali startups while preserving the conceptual meaning established in the supporting literature. In particular, the instrument avoided assumptions of highly developed patent portfolios, specialized IP departments, or sophisticated legal infrastructures and instead reflected forms of intangible asset protection and legal knowledge relevant to young firms operating in Somalia.

Standard PLS-SEM procedures were used to assess the reliability and validity of the measurement model. Indicator reliability was evaluated using outer loadings, internal consistency reliability using Cronbach’s alpha and composite reliability, convergent validity using average variance extracted (AVE), and discriminant validity using the Fornell–Larcker criterion and the Heterotrait–Monotrait ratio (Hair et al., 2024; Henseler et al., 2015).

### 3.6 Data analysis strategy

Data analysis was conducted using SmartPLS 4. The analytical procedure proceeded in two main stages. First, descriptive statistics were used to summarize respondent and startup characteristics, including gender, age, educational attainment, startup sector, startup age, and number of employees. Second, Partial Least Squares Structural Equation Modelling (PLS-SEM) was employed to assess the measurement and structural models and to test the proposed hypotheses.

The PLS-SEM analysis began with an assessment of the measurement model, including indicator reliability, internal consistency reliability, convergent validity, and discriminant validity. Indicators with outer loadings above 0.70 were considered satisfactory, whereas indicators with loadings between 0.40 and 0.70 could be retained when their removal did not improve composite reliability or average variance extracted (AVE) and when their theoretical relevance justified retention. Cronbach’s alpha and composite reliability values above 0.70 were considered acceptable, while AVE values above 0.50 indicated satisfactory convergent validity (Hair et al., 2019, 2024).

Discriminant validity was evaluated using the Heterotrait–Monotrait ratio (HTMT). HTMT values below 0.85 were considered evidence of satisfactory discriminant validity under a conservative criterion, while values below 0.90 were considered acceptable for conceptually related constructs (Hair et al., 2024). The Fornell–Larcker criterion was also examined as an additional assessment of discriminant validity.

Following confirmation of the measurement model, the structural model was assessed to examine the direct effects of intellectual property protection and legal awareness on startup growth and the moderating effect of legal awareness on the relationship between intellectual property protection and startup growth. The statistical significance of the structural paths was assessed using the bootstrapping procedure in SmartPLS 4 with 5,000 resamples. Path coefficients (*β*), standard errors, *t*-values, *p*-values, 95% confidence intervals, coefficients of determination (*R*^2^), and effect sizes (*f*^2^) were evaluated in accordance with established PLS-SEM reporting guidelines (Hair et al., 2024).

The moderating effect of legal awareness was assessed by estimating an interaction term between intellectual property protection and legal awareness (IPP × LA). A statistically significant positive interaction coefficient would indicate that the positive relationship between intellectual property protection and startup growth becomes stronger at higher levels of legal awareness. The structural model was specified as follows:

SG=β0+β₁IPP+β₂LA+β₃(IPP×LA)+ε

Where:

SG = Startup Growth.

IPP = Intellectual Property Protection.

LA = Legal Awareness.

IPP × LA = Interaction between Intellectual Property Protection and Legal Awareness.

*ε* = Error term.

The structural model was used to test the three hypotheses developed in Section 2 concerning the direct effects of intellectual property protection and legal awareness and the moderating role of legal awareness.

### 3.7 Ethical considerations

The study was approved by the Research Ethics Committee of SIMAD University with Approval No. EC000227, dated 15 February 2026. All participants were adults and provided written informed consent before taking part in the study. Participation was voluntary, and respondents were informed of the purpose of the study, their right to withdraw, and the confidentiality of their responses. No personal identification information was collected. Data were used only for academic research purposes and were stored securely. Results are reported in aggregate form to protect the identity of individual respondents and startups.

## 4 Results

### 4.1 Demographic information

Table 1 presents the demographic and startup characteristics of the 220 respondents included in the study. The profile covers respondents’ gender, age, educational attainment, startup sector, startup age, and number of employees. Reporting the complete distribution of these characteristics provides a comprehensive description of the sample and enables readers to assess the diversity and contextual relevance of the respondents included in the subsequent analysis.

**Table 1:** Demographic information.

Variable	Category	Frequency (*n*)	Percentage (%)
Gender	Male	136	61.8
	Female	84	38.2
Age group	18–25 years	64	29.1
	26–35 years	99	45.0
	36–45 years	51	23.2
	46 years and above	6	2.7
Education level	Secondary	32	14.5
	Diploma	38	17.3
	Bachelor’s degree	97	44.1
	Master’s degree	48	21.8
	Other	5	2.3
Startup sector	E-commerce	43	19.5
	Digital services	43	19.5
	Education/Training	27	12.3
	Retail/Trading	26	11.8
	Fintech	21	9.5
	Media/Creative	21	9.5
	Logistics	16	7.3
	Agribusiness	16	7.3
	Health services	7	3.2
Startup age	Less than 1 year	35	15.9
	1–3 years	95	43.2
	4–6 years	59	26.8
	7 years and above	31	14.1
Number of employees	1–5	99	45.0
	6–10	60	27.3
	11–20	44	20.0
	21 and above	17	7.7

As shown in Table 1, males constituted 61.8% of the sample, while females represented 38.2%. Respondents aged 26–35 years formed the largest age group (45.0%), and bachelor’s degree holders represented the largest educational category (44.1%). In terms of startup characteristics, firms operating for 1–3 years accounted for the largest proportion (43.2%), while 45.0% employed between one and five employees. E-commerce and digital services were the two largest sectors, each representing 19.5% of the sample, followed by education/training (12.3%) and retail/trading (11.8%). The startup sample represented a diverse range of business sectors. E-commerce and digital services were the two largest categories, each accounting for 19.5% of respondents, followed by education/training (12.3%), retail/trading (11.8%), fintech (9.5%), media/creative industries (9.5%), logistics (7.3%), agribusiness (7.3%), and health services (3.2%). The relatively strong representation of digital and service-oriented enterprises is particularly relevant because such businesses frequently depend on intangible assets, including brand identity, software, digital content, creative outputs, business know-how, and customer goodwill. These enterprises may also contribute to employment creation, market accessibility, digital inclusion, and the provision of new products and services. Nevertheless, startup growth should not automatically be equated with broader social or developmental values. Research on entrepreneurship within the Sustainable Development Goals framework indicates that commercially successful enterprises may differ considerably in the extent to which they pursue social objectives, serve disadvantaged populations, or address wider sustainability priorities (Li et al., 2024). Similarly, technological enterprises can contribute to public problem-solving when technological innovation is deliberately combined with public values and societal objectives rather than being assessed exclusively through commercial performance (Li et al., 2025).

Accordingly, the outcome examined in the present study is startup growth at the firm level, rather than social impact or inclusive development. The survey did not directly assess the quality of jobs created, wage conditions, employment security, affordability of products or services, or the extent to which individual startups contribute to the Sustainable Development Goals. The findings should therefore be interpreted as evidence concerning the relationship between intellectual property protection, legal awareness, and business growth, rather than as direct evidence of socially inclusive or sustainable development outcomes.

### 4.2 Descriptive statistics

Table 2 presents the descriptive statistics for the three main constructs: intellectual property protection, legal awareness, and startup growth. The mean values indicate moderate levels across all constructs. Intellectual property protection recorded the highest mean score (M = 3.251, SD = 1.073), followed by startup growth (M = 3.135, SD = 1.061) and legal awareness (M = 3.087, SD = 1.032). These results suggest that respondents moderately agreed that their startups engage in IP protection practices, possess legal awareness, and experience growth. The skewness and kurtosis values were within acceptable ranges, indicating that the data did not show serious distributional problems.

**Table 2:** Descriptive statistics.

Construct	Mean	SD	Minimum	Maximum	Skewness	Kurtosis
Intellectual property protection	3.251	1.073	1.00	5.00	−0.129	−0.929
Startup growth	3.135	1.061	1.00	5.00	−0.012	−0.883
Legal awareness	3.087	1.032	1.00	5.00	−0.035	−0.819

Overall, the descriptive results suggest that Somali startups in the sample demonstrate moderate levels of IP protection, legal awareness, and perceived growth. This reflects a developing startup ecosystem in which entrepreneurs are increasingly aware of intangible asset protection and legal issues, but these practices are not yet strongly institutionalized.

### 4.3 Measurement model

The measurement model was assessed before testing the structural relationships. Table 3 presents the reliability and convergent validity results for intellectual property protection, legal awareness, and startup growth. Cronbach’s Alpha value ranged from 0.898 to 0.923, exceeding the recommended threshold of 0.70. Composite Reliability values ranged from 0.925 to 0.942, also exceeding the recommended level. The AVE values were above 0.50 for all constructs, confirming convergent validity. The item loading ranges were also acceptable: 0.854–0.893 for intellectual property protection, 0.828–0.853 for startup growth, and 0.834–0.880 for legal awareness.

**Table 3:** Measurement model results.

Construct	Item	Measurement item	Outer loading	Cronbach’s *α*	Composite reliability	AVE
Intellectual Property Protection	IPP1	Our startup takes appropriate steps to protect its intellectual property, including its brand, business name, software, designs, creative works, or other intangible assets.	0.871	0.923	0.942	0.765
	IPP2	Our startup uses appropriate measures to establish and protect ownership of its intellectual property and other intangible assets.	0.874			
	IPP3	Our startup uses formal registration, contractual arrangements, or confidentiality measures where appropriate to protect its intellectual property.	0.879			
	IPP4	Our startup takes measures to reduce the risk of unauthorized use, copying, or infringement of its intellectual property.	0.893			
	IPP5	Our startup uses intellectual property protection as part of its strategy to support competitiveness and business development.	0.854			
Startup Growth	SG1	Our startup has increased its number of customers during the past year.	0.840	0.898	0.925	0.712
	SG2	Our startup has improved its sales or revenue performance during the past year.	0.844			
	SG3	Our startup has expanded its products or services during the past year.	0.828			
	SG4	Our startup has created additional employment opportunities during the past year.	0.853			
	SG5	Our startup has expanded into new markets or customer segments during the past year.	0.852			
Legal Awareness	LA1	I understand the basic legal requirements relevant to protecting and operating a startup in Somalia.	0.864	0.911	0.934	0.737
	LA2	I understand the legal procedures for registering and protecting a business name, brand, product, or other intellectual property.	0.880			
	LA3	I understand the legal requirements related to ownership, contracts, confidentiality, and licensing that are relevant to my startup.	0.859			
	LA4	I am aware of the legal risks associated with intellectual property infringement, contractual disputes, and unauthorized use of business information.	0.857			
	LA5	I know where and how to obtain appropriate legal advice when my startup faces an important legal or intellectual property issue.	0.834			

The measurement model was assessed before examining the structural relationships. Table 3 reports the individual outer loadings for the 15 observed indicators together with Cronbach’s alpha, composite reliability, and average variance extracted (AVE) for each latent construct. All indicator loadings exceeded the recommended threshold of 0.70, ranging from 0.854 to 0.893 for intellectual property protection, 0.828 to 0.853 for startup growth, and 0.834 to 0.880 for legal awareness. Cronbach’s alpha values ranged from 0.898 to 0.923, while composite reliability ranged from 0.925 to 0.942. AVE values ranged from 0.712 to 0.765, exceeding the recommended threshold of 0.50. These results support satisfactory indicator reliability, internal consistency reliability, and convergent validity.

### 4.4 Discriminant validity

Discriminant validity was assessed using the Fornell–Larcker criterion and HTMT ratios. As shown in Table 4, the square root of AVE for each construct was higher than its correlations with other constructs. For example, the square root of AVE for intellectual property protection was 0.874, which was higher than its correlations with startup growth (0.345) and legal awareness (0.187). Similarly, the square root of AVE for startup growth was 0.844, and the square root of AVE for legal awareness was 0.859, both exceeding their respective inter-construct correlations.

**Table 4:** Fornell–Larcker criterion.

Construct	IPP	SG	LA
Intellectual property protection	0.874		
Startup growth	0.345	0.844	
Legal awareness	0.187	0.446	0.859

The Fornell–Larcker results indicate that each construct shared more variance with its own indicators than with other constructs in the model. This supports the discriminant validity of intellectual property protection, legal awareness, and startup growth.

Table 5 presents the HTMT results. All HTMT values were below the conservative threshold of 0.85. The highest HTMT value was between startup growth and legal awareness (0.493), followed by intellectual property protection and startup growth (0.377), and intellectual property protection and legal awareness (0.203). These results further confirm that the constructs are empirically distinct.

**Table 5:** Heterotrait–Monotrait ratio.

Construct pair	HTMT
IPP–SG	0.377
IPP–LA	0.203
SG–LA	0.493

Taken together, the Fornell–Larcker and HTMT results confirm discriminant validity. Therefore, the constructs were sufficiently distinct and suitable for structural model analysis.

### 4.5 Structural model

After confirming the reliability, convergent validity, and discriminant validity of the measurement model, the structural model was assessed. The model explained 27.1% of the variance in startup growth, with an adjusted *R*^2^ of 26.1% (Table 6). This indicates that intellectual property protection, legal awareness, and their interaction explain a meaningful proportion of startup growth, especially considering that startup growth may also be influenced by other factors such as access to finance, founder experience, market competition, digital capability, and institutional support.

**Table 6:** Coefficient of determination.

Endogenous construct	*R* ^2^	Adjusted *R*^2^
Startup growth	0.271	0.261

Table 7 presents the hypothesis-testing results. Intellectual property protection had a positive and significant effect on startup growth (*β* = 0.271, SE = 0.055, *t* = 4.946, *p* < 0.001). The 95% confidence interval [0.163, 0.378] did not include zero, confirming the significance of the relationship. The effect size was small to moderate (*f*^2^ = 0.097). Therefore, H1 was supported.

**Table 7:** Structural model and hypothesis testing.

H	Path	*β*	SE	*t*-value	*p*-value	95% CI	*f* ^2^	Decision
H1	IPP → SG	0.271	0.055	4.946	<0.001	[0.163, 0.378]	0.097	Supported
H2	LA → SG	0.394	0.058	6.839	<0.001	[0.274, 0.498]	0.204	Supported
H3	IPP × LA → SG	0.029	0.053	0.554	0.580	[−0.075, 0.134]	0.001	Not supported

Legal awareness also had a positive and significant effect on startup growth (*β* = 0.394, SE = 0.058, *t* = 6.839, *p* < 0.001). The confidence interval [0.274, 0.498] was fully positive, confirming the significance of the effect. The effect size was 0.204, indicating that legal awareness had a stronger effect on startup growth than intellectual property protection. Therefore, H2 was supported.

However, the interaction effect between intellectual property protection and legal awareness was not statistically significant (*β* = 0.029, SE = 0.053, *t* = 0.554, *p* = 0.580). The confidence interval [−0.075, 0.134] included zero, and the effect size was negligible (*f*^2^ = 0.001). Therefore, H3 was not supported.

The moderation analysis did not provide support for H3. The interaction between intellectual property protection and legal awareness had a small positive coefficient (*β* = 0.029), but the effect was statistically non-significant (*p* = 0.580). Moreover, the 95% confidence interval extended from −0.075 to 0.134 and therefore included zero, while the interaction effect size was negligible (*f*^2^ = 0.001). Taking together, these results indicate that changes in legal awareness do not meaningfully alter the strength of the relationship between intellectual property protection and startup growth in the present sample. Legal awareness therefore appears to contribute to startup growth primarily through its significant direct effect rather than by amplifying the effect of intellectual property protection (Figure 1).

**Figure 1:**
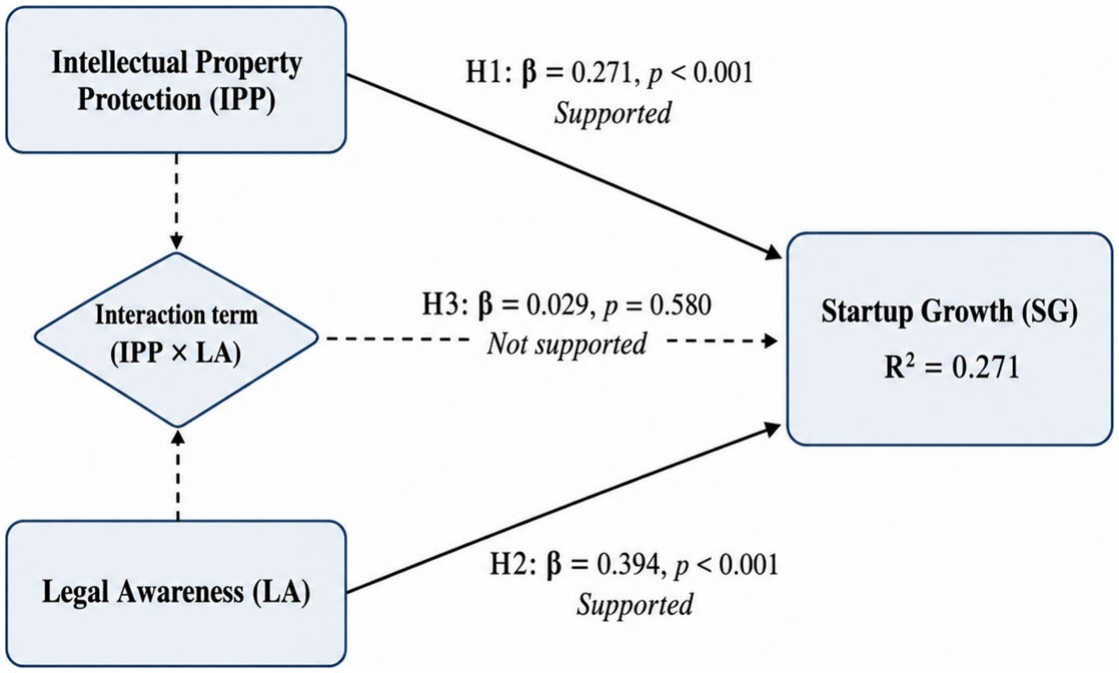
PLS-SEM structural model and hypothesis results. Solid arrows represent the direct effects, while the dashed arrow represents the interaction effect used to test moderation. *p* < 0.001; n.s. = not significant. The interaction effect of IPP × LA on SG was not statistically significant.

## 5 Discussion

This study examined the effects of intellectual property protection and legal awareness on startup growth in Somalia and tested whether legal awareness strengthens the relationship between intellectual property protection and startup growth. The findings show that intellectual property protection has a positive and significant effect on startup growth. Legal awareness also has a positive and significant direct effect on startup growth, and its effect is stronger than that of intellectual property protection. However, the interaction between intellectual property protection and legal awareness is not statistically significant. These results suggest that both IP protection and legal awareness are important for startup growth in Somalia, but legal awareness functions more as an independent growth-enabling capability than as a moderator of the IP protection–startup growth relationship.

The positive effect of intellectual property protection on startup growth supports the Resource-Based View. RBV argues that firms can achieve superior performance when they possess valuable, rare, difficult-to-imitate, and non-substitutable resources. In the startup context, intellectual property can function as such a strategic resource because trademarks, copyrights, industrial designs, software, trade secrets, business names, and brand identity may help firms protect innovation, reduce imitation, and differentiate themselves from competitors. This finding is consistent with Power and Reid (2021) who showed that different types of intellectual property can influence startup performance, although the effect varies across IP instruments. It also supports Grimaldi et al. (2021), who argued that IP strategies help firms protect knowledge and strengthen their innovation position when IP is used strategically rather than treated only as a legal formality.

The positive firm-level effect identified in this study should not, however, be interpreted as evidence that stronger intellectual property protection is always socially or developmentally optimal. Intellectual property policy involves a balance between creating incentives for innovation and ensuring that valuable knowledge and technologies can be accessed, diffused, and adapted. This balance may be particularly important in developing economies such as Somalia, where startups can benefit from protecting locally developed intangible assets while also depending heavily on imported technology, knowledge transfer, and adaptive innovation. Therefore, the present findings support strengthening effective and accessible mechanisms for protecting entrepreneurial assets rather than adopting an indiscriminately restrictive intellectual property regime. Consistent with the broader sustainable-development literature, IP policy should seek to combine incentives for innovation with competition, technology diffusion, affordability, and wider societal access (Khouilla and Bastidon, 2024; Jee et al., 2026).

The result also aligns with evidence from developing and emerging economies. Amankwah-Amoah and Medase (2023) found that intellectual property can help firms extract innovation value in sub-Saharan Africa, although its effect depends on the type of IP and the institutional environment. Similarly, Adomako and Tran (2025) linked intellectual property rights protection to sustainable innovation performance, showing that IP protection can support firm-level outcomes when firms can use legal protection alongside complementary resources. The present study extends this literature by providing empirical evidence from Somalia, a fragile and under-researched startup context. The findings indicate that even where formal IP institutions are still developing, startups that protect their intangible assets are more likely to report stronger growth.

This finding is particularly relevant for Somali startups because many operate in sectors where intangible assets are central to competitiveness. E-commerce, digital services, media, education technology, consultancy, fintech, and creative businesses often depend on business names, logos, digital platforms, software, creative content, designs, and customer trust. Protecting these assets can support growth by strengthening brand identity, improving market credibility, and reducing the risk of imitation. This interpretation is consistent with, Rieger et al. (2025) who found that trademark applications may serve as early legitimacy signals in attracting venture capital seed funding. It is also consistent with Taglialatela and Barontini (2024), who showed that patent-related protection may be associated with sales improvement, although not necessarily profitability. These studies confirm that IP protection can support growth, but its value depends on how entrepreneurs use it within their business strategy.

The positive relationship between intellectual property protection and startup growth should nevertheless be interpreted within the boundaries of the outcome measured in this study. Startup growth in the present analysis concerns firm-level developments such as sales, customer base, market reach, product or service expansion, operational capacity, and competitive position. The findings do not establish whether such growth generates secure and adequately remunerated employment, improves the affordability of goods and services, or contributes directly to specific Sustainable Development Goals. These are related but conceptually distinct outcomes. Research on entrepreneurship and the Sustainable Development Goals indicates that commercially successful enterprises vary substantially to the extent to which they pursue social objectives and generate broader public value (Li et al., 2024). Similarly, technological enterprises may contribute to integrated public problem-solving when commercial and technological capabilities are deliberately aligned with societal needs and public objectives (Li et al., 2025). Future research in Somalia should therefore examine whether IP-supported startup growth also translates into employment quality, affordable and accessible products and services, social innovation, and measurable contributions to sustainable development.

The second major finding is that legal awareness has a positive and significant effect on startup growth. This result is theoretically important because legal awareness had a stronger effect on startup growth than intellectual property protection. This suggests that legal knowledge is not merely a supporting factor but a business capability. Entrepreneurs who understand contracts, ownership rights, registration procedures, licensing, confidentiality, infringement risks, and dispute prevention may be better able to reduce uncertainty, protect business relationships, and make informed growth decisions. This finding supports Resource Orchestration Theory, which argues that resources do not automatically generate performance; instead, managers must structure, bundle, and leverage resources effectively before they create value (D’Oria et al., 2021).

From a resource orchestration perspective, legal awareness helps explain how entrepreneurs convert legal knowledge into practical business decisions. A startup may possess a brand, software, product design, creative work, or trade secret, but these assets may not produce growth if the entrepreneur cannot document ownership, use written agreements, prevent infringement, protect confidential information, or negotiate licensing and partnership arrangements. Legal awareness therefore operates as a managerial capability that helps entrepreneurs use legal and intangible resources more effectively. This interpretation is consistent with Khawand et al. (2024) who emphasized the importance of IP knowledge and strategy in SME decision-making. It also aligns with Sukania et al. (2025) who linked legal protection and legal awareness to SME performance and compliance behavior.

The Somali institutional context strengthens the importance of this finding. Somalia’s legal and regulatory environment is still developing, and startups may face difficulties in accessing predictable enforcement mechanisms. The International Trade Administration (2024) notes that Somali courts face limitations in resolving intellectual property claims because of broader judicial challenges. Similarly, Chambers and Partners (2026) indicates that Somalia does not have specialized intellectual property courts and that IP disputes are generally handled through ordinary civil mechanisms. In such a setting, legal awareness may help entrepreneurs act preventively before disputes arise. Entrepreneurs who understand relevant legal requirements, registration procedures, contracts, confidentiality, ownership, and available legal assistance may be better positioned to reduce legal risk even where formal enforcement remains limited.

The third finding, the non-significant moderating effect of legal awareness, is one of the most important contributions of the study. The study expected legal awareness to strengthen the relationship between intellectual property protection and startup growth. However, the results do not support this expectation. This means that although legal awareness directly supports startup growth, it does not significantly increase the effect of IP protection on growth. In practical terms, legally aware entrepreneurs may grow their businesses through better contracts, compliance, partnership management, risk reduction, and dispute prevention, not only through stronger use of IP protection.

The significant direct effect of legal awareness also indicates that legal knowledge relevant to startup growth extends beyond intellectual property protection itself. In particular, awareness of basic legal requirements for operating and protecting a startup, ownership arrangements, commercial contracts, confidentiality, licensing, legal and infringement risks, and access to appropriate legal advice may help entrepreneurs make more informed business decisions and reduce legal uncertainty. These capabilities can support the continuity and development of startups even when they do not directly involve formal intellectual property protection. Thus, the finding suggests that legal awareness should be understood as a broader entrepreneurial capability that supports legally informed decision-making and business risk management.

Several explanations may account for this result. First, Somalia’s IP institutional framework may not yet be strong enough for legal awareness to amplify the commercial value of IP protection. Entrepreneurs may understand the importance of IP rights, but if registration systems, enforcement processes, legal services, and dispute-resolution mechanisms remain limited or costly, awareness alone may not be enough to make IP protection more growth-enhancing. This interpretation is consistent with Institutional Theory, which argues that the value of firm resources is shaped by the wider legal and regulatory environment. In stronger institutional settings, legal awareness may help entrepreneurs use IP protection more effectively; in fragile institutional settings, the effect may be constrained by weak enforcement and limited legal infrastructure.

Second, many Somali startups may rely on informal or semi-formal protection mechanisms alongside formal IP protection. In fragile markets, entrepreneurs may protect business value through reputation, personal networks, customer loyalty, community trust, first-mover advantage, secrecy, and informal negotiation. These mechanisms may reduce the extent to which the IP protection–growth relationship depends on formal legal awareness. This means that IP protection may still contribute to growth, but legal awareness may not necessarily strengthen that specific relationship because entrepreneurs use several other mechanisms to protect and expand their businesses.

Third, the result may reflect the early-stage profile of many startups in the sample. Many respondents represented young and small startups, particularly businesses aged one to 3 years and firms with one to five employees. Such startups may not yet have advanced IP strategies, licensing agreements, investor negotiations, patent portfolios, or formal enforcement experience. For these firms, IP protection may mainly involve basic practices such as protecting business names, logos, software, designs, and creative works. Legal awareness may be useful for broader business management, but it may not yet operate as a strong condition that increases the growth value of IP protection.

The findings refine the three theories used in this study. RBV explains why IP protection matters: protected intangible assets can support differentiation, imitation prevention, legitimacy, and competitive advantage. Resource Orchestration Theory explains why legal awareness matters: entrepreneurs need legal and managerial capability to use resources effectively. Institutional Theory explains why the moderation hypothesis was not supported: in a fragile institutional environment, legal awareness may not be sufficient to strengthen the effect of IP protection unless legal institutions are accessible, predictable, and enforceable. Therefore, the study shows that startup growth in Somalia depends not only on possessing IP-related resources, but also on entrepreneurs’ legal capabilities and the institutional environment in which those capabilities are used.

The findings also have practical implications. Startup support programs in Somalia should not focus only on finance, marketing, and incubation. They should also include practical legal-awareness training covering startup legal requirements, business and intellectual property registration, ownership, contracts, licensing, confidentiality, legal-risk management, and access to appropriate legal advice. Universities, incubators, business associations, and legal professionals can support startups by offering legal clinics, simplified IP guidance, contract templates, and practical training. Government institutions can also strengthen startup growth by making IP registration more accessible, improving public guidance, digitalizing registration procedures, and strengthening commercial dispute-resolution mechanisms.

Overall, this study contributes to entrepreneurship and intellectual property literature by providing evidence from Somalia, a fragile and under-researched context. It confirms that both IP protection and legal awareness directly support startup growth. However, it also shows that legal awareness should not automatically be treated as a moderator of the IP protection-growth relationship. In the Somali context, legal awareness appears to operate as an independent legal-managerial capability that supports growth through broader business practices. Future research should examine whether this relationship differs by sector, startup age, type of IP protection, access to finance, innovation capability, institutional trust, or the strength of formal legal enforcement.

### 5.1 Theoretical implications

The findings make several theoretical contributions to the literature on intellectual property, entrepreneurship, and startup development. First, the positive relationship between intellectual property protection and startup growth reinforces the Resource-Based View by showing that protected intangible assets can function as strategically valuable resources even in an institutionally fragile entrepreneurial environment. Existing studies have demonstrated that trademarks, patents, copyrights, designs, and related IP strategies can influence startup and SME performance, although their effects differ across types of IP and institutional conditions (Grimaldi et al., 2021). The present findings extend this argument to Somalia and indicate that the strategic value of intangible assets is not confined to highly developed IP systems.

Second, the comparatively stronger direct effect of legal awareness contributes to Resource Orchestration Theory. The result suggests that legal knowledge should be treated as an entrepreneurial capability rather than merely as background knowledge supporting formal IP protection. Resource Orchestration Theory emphasizes that strategic resources create value only when managers can structure, bundle, and leverage them effectively (D’Oria et al., 2021). In the present context, awareness of basic legal requirements, ownership, registration procedures, contracts, licensing, confidentiality, infringement risks, and access to legal assistance may enable entrepreneurs to reduce uncertainty and organize business relationships more effectively. The findings therefore extend resource-orchestration reasoning by identifying legal awareness as a capability through which entrepreneurs may support growth independently of their formal IP-protection activities.

Third, the non-significant moderation effect refines the theoretical relationship between resources and complementary capabilities. The study originally expected legal awareness to increase the growth value of intellectual property protection. Instead, legal awareness produced a significant direct contribution to startup growth while its interaction with IP protection was negligible. This finding indicates that complementary capabilities should not automatically be assumed to amplify the effects of strategic resources. Their role may depend on the institutional environment in which resources are deployed. Institutional Theory helps explain this result because Somalia’s developing IP enforcement and dispute-resolution infrastructure may constrain the extent to which greater legal knowledge can enhance the commercial value of formal IP protection (Chambers and Partners, 2026).

For scholars, these findings suggest that future research should distinguish between the direct, complementary, and contingent roles of legal capabilities rather than assuming that legal awareness necessarily functions as a moderator. The results also support more context-sensitive testing of RBV and Resource Orchestration Theory in fragile and emerging economies, where the value of organizational resources may depend substantially on institutional quality. Comparative studies across stronger and weaker IP regimes could establish whether legal awareness becomes a significant moderator when registration, enforcement, and commercial dispute-resolution mechanisms are more predictable.

### 5.2 Practical and policy implications

The findings also have implications for startup founders and managers. The significant effects of both IP protection and legal awareness indicate that entrepreneurs should treat legal capability and intangible-asset management as components of business strategy rather than as activities undertaken only after a dispute occurs. Founders and managers should identify important intangible assets at an early stage, clarify ownership, use appropriate confidentiality and contractual safeguards, consider formal registration where available and commercially appropriate, and develop sufficient legal knowledge to manage licensing, partnerships, infringement risks, and commercial agreements. This is consistent with prior research showing that IP generates greater value when it is incorporated into broader strategic decision-making rather than treated solely as a formal legal instrument (Grimaldi et al., 2021; Khawand et al., 2024).

For policymakers, the stronger direct effect of legal awareness suggests that strengthening Somalia’s startup ecosystem requires more than expanding formal IP-registration mechanisms. Public institutions should complement registration services with accessible legal information, simplified procedural guidance, digitalized services, awareness campaigns, and mechanisms that enable young firms to obtain affordable legal assistance. At the same time, the absence of a significant moderation effect cautions against assuming that entrepreneurs’ education alone can overcome institutional weaknesses. Improvements in enforcement predictability, commercial dispute resolution, institutional coordination, and access to legal remedies remain important if formal IP protection is to generate greater commercial value (International Trade Administration, 2024; Chambers and Partners, 2026).

Accordingly, startup-support programs should complement intellectual property initiatives with practical legal-awareness training covering the legal requirements for startup operation and protection, ownership and contractual arrangements, confidentiality, licensing, legal-risk management, and pathways for obtaining appropriate legal advice. Universities, incubators, accelerators, business associations, and entrepreneurship-support organizations could provide practical legal guidance, simplified registration information, basic contract and confidentiality resources, and referral mechanisms to appropriate legal professionals. Such interventions may help entrepreneurs identify legal risks at an early stage and incorporate legal considerations into routine business decision-making rather than seeking legal assistance only after disputes arise.

For universities, incubators, accelerators, business associations, and entrepreneurship-support organizations, the results support integrating practical legal capability into conventional startup-support programs. Training should extend beyond general descriptions of intellectual property rights to include identifying protectable assets, ownership documentation, confidentiality agreements, licensing, basic contract management, infringement prevention, and pathways for obtaining professional legal advice. Universities and legal institutions could establish startup legal clinics, while incubators and business associations could provide standardized guidance materials, introductory contract templates, referral systems, and periodic IP-awareness sessions. Such initiatives may be particularly valuable for young and small firms that lack dedicated legal personnel.

For legal professionals and organizations providing professional services, the findings indicate an opportunity to develop startup-oriented legal services that are preventive, affordable, and accessible. Traditional legal services that become available primarily after disputes arise may be poorly suited to early-stage firms. Lawyers and IP practitioners can instead support startups through preventive legal audits, ownership clarification, contract review, trademark and other registration guidance, confidentiality arrangements, licensing advice, and dispute-prevention strategies. This is particularly relevant in Somalia, where formal enforcement limitations increase the importance of documenting rights and managing legal risks before conflicts emerge.

Finally, these implications should be interpreted considering the broader public-value considerations discussed in this study. Strengthening IP protection should not be understood simply by maximizing exclusivity. Startup policy should balance incentives to protect entrepreneurial innovation with the diffusion of knowledge, competition, accessibility, and wider development objectives. The policy goal is therefore an IP environment in which startups can protect legitimate intangible assets while remaining able to access, adapt, and build upon knowledge needed for innovation and sustainable development.

## 6 Conclusion

This study examined the effects of intellectual property protection and legal awareness on startup growth in Somalia. The findings show that both intellectual property protection and legal awareness have positive and significant direct effects on startup growth. However, legal awareness does not significantly moderate the relationship between intellectual property protection and startup growth. This indicates that legal awareness is better understood as an independent growth-enabling capability rather than as a factor that strengthens the effect of IP protection.

The study contributes to entrepreneurship and IP management literature by providing evidence from Somalia, a fragile and under-researched institutional context. It shows that protecting intangible assets and improving entrepreneurs’ legal awareness are both important for startup development. However, the findings also suggest that legal awareness alone may not be sufficient to maximize the growth value of IP protection unless the broader legal and institutional environment becomes more accessible, predictable, and enforceable. Future research should examine additional factors such as access to finance, digital capability, innovation capacity, institutional trust, and sectoral differences using longitudinal or mixed-method designs.

### 6.1 Limitations and future research

This study has several limitations that should be considered when interpreting the findings. First, the study employed purposive non-probability sampling because a complete national sampling frame specifically identifying Somali startups was unavailable. Although the sample included knowledgeable founders, owners, managers, and senior employees from different sectors and geographic locations, it cannot be regarded as statistically representative of the entire Somali startup population. Consequently, the findings should be interpreted as analytical evidence from the participating startups rather than as population-level estimates. Future research could improve external validity by using probability-based sampling where a reliable startup registry becomes available or by constructing stratified samples across regions, sectors, and startup-development stages.

Second, the cross-sectional research design captured intellectual property protection, legal awareness, and startup growth at a single point in time. The observed relationships therefore indicate statistical associations rather than definitive causal effects. For example, while stronger intellectual property protection and legal awareness were associated with higher perceived startup growth, the design cannot establish whether these factors caused subsequent growth or whether growing startups developed stronger legal and IP capabilities. Longitudinal studies that follow startups over time provide stronger evidence regarding temporal ordering and causal mechanisms. Experimental or quasi-experimental approaches could also examine whether targeted IP or legal awareness interventions produce measurable changes in startup outcomes.

Third, the study relied on self-reported and perceptual measures, including respondents’ assessments of startup growth. Although this approach was appropriate because many young and privately held startups may not maintain standardized audited financial records or may be reluctant to disclose commercially sensitive information, self-reporting may introduce common-method, recall, or social-desirability bias. These potential biases may influence the magnitude of the estimated relationships. Future studies should combine perceptual measures with objective indicators, where available, such as sales growth, employment growth, market expansion, investment raised, profitability, or formal IP-registration records. Multi-source data collected from founders, employees, legal advisers, and administrative records could further strengthen measurement validity.

Fourth, the explanatory model was deliberately parsimonious and focused on intellectual property protection, legal awareness, and startup growth. Although the model explained 27.1% of the variance in startup growth, a substantial proportion of variation remains attributable to other factors not examined in the study. Access to finance, founder experience, innovation capability, digital capability, market competition, institutional trust, government support, and sectoral conditions may independently or jointly influence startup growth. Future research could extend the structural model by incorporating these variables as additional predictors, mediators, moderators, or control variables and by comparing alternative models across startup sectors and institutional environments.

Fifth, the study examined firm-level startup growth rather than the broader socioeconomic consequences of startup expansion. The questionnaire did not directly measure job quality, wages, employment security, affordability or accessibility of products and services, social innovation, environmental outcomes, or contributions to specific Sustainable Development Goals. Accordingly, the positive relationships identified in this study should not be interpreted as evidence that startup growth automatically produces inclusive or sustainable development outcomes. Future research should connect intellectual property protection and legal capability with broader measures of public value, including decent employment, affordability, social inclusion, technology diffusion, and sustainable-development performance.

Finally, the non-significant moderating effect of legal awareness should be interpreted within Somalia’s specific institutional setting. The result suggests that legal awareness operates primarily as a direct growth-related capability in the present sample, but this pattern may differ in environments with stronger IP enforcement, more accessible legal services, or more mature startup ecosystems. Comparative research across countries or institutional settings would help determine whether the moderating role of legal awareness is context dependent. Mixed-method studies could also complement the quantitative findings by exploring how entrepreneurs use legal knowledge and IP protection in day-to-day strategic decision-making.

## Data Availability

The raw data supporting the conclusions of this article will be made available by the authors, without undue reservation.
